# Prevalence of chloroquine resistance alleles among *Plasmodium falciparum* parasites in countries affected by malaria disease since change of treatment policy: a systematic review protocol

**DOI:** 10.1186/s13643-018-0780-z

**Published:** 2018-07-27

**Authors:** Moses Ocan, Dickens Akena, Sam Nsobya, Moses R. Kamya, Richard Senono, Alison Annet Kinengyere, Ekwaro A. Obuku

**Affiliations:** 1 Department of Pharmacology & Therapeutics, Makerere University, P.O. Box 7072, Kampala, Uganda; 2 Department of Psychiatry, Makerere University, P.O. Box 7072, Kampala, Uganda; 3 Department of Medical Microbiology, Makerere University, P.O. Box 7072, Kampala, Uganda; 4 Department of Medicine, Makerere University, P.O. Box 7072, Kampala, Uganda; 5 Africa Centre for Knowledge Translation and Systematic Reviews, College of Health Sciences, Department of Medicine, Makerere University, Kampala, Uganda; 6 Infectious Disease Institute, Makerere University, P. O. Box 22418, Kampala, Uganda; 7 Albert Cook library, Makerere University, P.O. Box 7072, Kampala, Uganda

**Keywords:** Chloroquine, Re-emergence, Sensitivity, *Plasmodium falciparum*, Policy

## Abstract

**Background:**

Malaria remains one of the leading causes of morbidity and mortality in most low- and middle-income countries. Chloroquine is a previously cheap and effective antimalarial agent whose loss to resistance resulted in more than doubling of malaria-related mortality in malaria-endemic countries. Recently, chloroquine sensitivity is re-emerging among *Plasmodium falciparum* parasites which gives hope for malaria control and treatment efforts globally. The aim of the current review is to establish the prevalence of chloroquine resistance alleles among *P*. *falciparum* parasites in malaria-endemic areas after change in malaria treatment policy.

**Methods/design:**

The articles will be obtained from search of MEDLINE via PubMed, SCOPUS, and EMBASE data bases. The Mesh terms will be used in article search. Boolean operators (“AND,” “OR”) will be used in article search. The article search will be done independently by two librarians. The PRISMA-P statement will be used to guide the conduct and reporting of the systematic review. STREGA guideline will be used in developing data abstraction form for the review. Data abstraction will be done by two independent reviewers, Kappa statistic will be calculated, and any discrepancies resolved by discussion. Data analysis will be done using STATA *ver* 13.0. The level of heterogeneity in the articles will be established by using the *I*^2^-statistic. Publication bias will be assessed using funnel plot. Random effects analysis will be used.

**Discussion:**

The review seeks to establish the extent of chloroquine resistance reversal in malaria-endemic countries. The evidence generated from this review will help guide policy makers on the potential re-emerging role of chloroquine in malaria treatment.

**Systematic review registration:**

The systematic review protocol has been registered in PROSPERO with registration number CRD42018083957.

**Electronic supplementary material:**

The online version of this article (10.1186/s13643-018-0780-z) contains supplementary material, which is available to authorized users.

## Background

Malaria treatment has undergone multiple changes over time due especially to the development of resistance against antimalarial agents. *Plasmodium* parasites, the causative agents of malaria, have managed to develop resistance to almost all drugs introduced in malaria treatment to date [1]. Over time, the old antimalarial agents for which *Plasmodium* parasites develop resistance are replaced with other effective agents in malaria treatment. However, recent studies are reporting re-emergence of parasite sensitivity to some medicines that had previously been withdrawn in malaria treatment due to high levels of resistance such as chloroquine [2]. Such reports provide hope for malaria treatment especially now that resistance has emerged to the only available and effective antimalarial agents, the artemisinin [3]. However, there is still uncertainty regarding the extent to which *Plasmodium* parasites have regained sensitivity to chloroquine in most malaria-endemic countries globally. In addition, how *Plasmodium* parasites will react to the re-introduction of chloroquine in malaria treatment in areas where resistance had previously reached fixation levels is not known. Some studies have suggested that if chloroquine is to be re-introduced in malaria treatment, it needs to be used in combination with another agent to help minimize the risk of resistance development [4]. This lack of evidence on the current extent of global chloroquine resistance could potentially contribute to the failure in re-introducing chloroquine in malaria treatment. A recent narrative review on chloroquine resistance reversal among *Plasmodium falciparum* parasites by Frosch et al. [2] focused only on African countries and did not provide aggregate extent of chloroquine resistance reversal.

Studies have investigated possibility of chemically altering chloroquine resistance which is mainly through efflux of the drug from parasite food vacuole. Chemicals like verapamil when combined with chloroquine have shown encouraging results in altering chloroquine resistance in *Plasmodium* parasites [5]. However, these attempts still use known medicines which are used for other conditions and thus potentially expose patients to the risk of toxicity [5]. This systematic review thus seeks to establish the extent of chloroquine resistance reversal in malaria-affected countries and the associated factors and assess the implications of this reversal on potential re-introduction of chloroquine in malaria treatment.

## Methods/design

The review protocol has been developed following the PRISMA-P 2015 statement [6] (Additional file 1).

### Review question

What is the prevalence of *Pfcrt* K76T and *Pfmdr1* N86Y alleles among *Plasmodium falciparum* parasites in malaria- affected countries since change in treatment policy from chloroquine to artemisinin agents?

### Systematic review objectives

The review will meet the following research objectives: (1) to establish the prevalence of chloroquine resistance alleles, *Pfcrt* K76T and *Pfmdr1* N86Y, among *P*. *falciparum* parasites in malaria-affected regions of the world after change in malaria treatment policy; (2) to determine the trends in the prevalence of chloroquine resistance alleles, *Pfcrt* K76T and *Pfmdr1* N86Y, among *P*. *falciparum* parasites in malaria-affected regions globally since the change in malaria treatment policy; and (3) to determine factors associated with occurrence of chloroquine resistance alleles among *P*. *falciparum* parasites in malaria-affected regions globally since change in malaria treatment policy.

### Registration of protocol

The review protocol has been registered in the International Prospective Register of Systematic Reviews (PROSPERO), registration number CRD 42018083957.

## Eligibility criteria

### Inclusion criteria

Articles will be screened for inclusion to the review and included articles have to fulfill the following requirements: (i) published from 1990–to-date in peer-reviewed journals; (ii) report on *P*. *falciparum* chloroquine molecular resistance (polymorphisms), *Pfcrt* K76T, and *Pfmdr1* N86Y; (iii) report both molecular (*Pfcrt* K76T and *Pfmdr1* N86Y) and phenotypic (IC_50_ or RSA_0-3 h_) resistance; (iv) polymorphisms in *P*. *falciparum* parasites assessed using restriction fragment length polymorphism (RFLP-PCR) or sequencing methods; (v) report genotypic chloroquine resistance (*Pfcrt* K76T and *Pfmdr1* N86Y) in *Plasmodium falciparum* parasites; (vi) studies done in countries in which malaria treatment policy was changed from chloroquine to artemisinin agents; (vii) report prevalence of *Pfcrt* K76T and *Pfmdr1* N86Y on day zero samples from clinical trial studies; and (viii) report on phenotypic (drug sensitivity) resistance of *P*. *falciparum* parasites.

### Exclusion criteria

Articles with the following characteristics will be excluded from the review: (i) report on resistance in non *P*. *falciparum* malaria (*P*. *vivax*, *P*. *malariae*, *P*. *knowleski*) and (ii) report on *P*. *falciparum* genotypic resistance prior to change in malaria treatment policy from chloroquine to artemisinin agents.

### Language

There will be no language restriction applied in the review.

### Search strategy

Published articles on prevalence of *Pfcrt* K76T, *Pfmdr1* N86Y, and parasite sensitivity (phenotype) to chloroquine from the time when the policy was changed to artemisinin agents will be searched from MEDLINE via PubMed, SCOPUS, EMBASE, and LILACS/VHL data bases. The search will cover a period from 1990-to-date and will include all countries affected by malaria disease. Bibliography of full-text articles will be searched for potential articles to be included in the review. Contact with authors and malaria researchers will be done to obtain any additional articles.

### Search terms

Boolean operators “OR” and “AND” will be used during article search. The following search terms were developed from reviewing articles published in peer-reviewed journals on chloroquine resistance. The following search terms will be used: “chloroquine,” “resistance,” malaria, fever, “reversal,” “prevalence,” “change,” “*Plasmodium falciparum*,” “*Pfcrt* K76T,” “*Pfmdr1* N86Y,” “resistance genes,” “resistance alleles,” “tropical countries,” “globally.” The search string will be developed using the above terms (Additional file 2).

### Article search

Two experienced librarians (AK and SR) will independently search for the articles from established databases. Articles from the two independent searches will then be merged in EndNote software and duplicates removed.

### Article review

Three independent reviewers will screen the articles using a pre-set criteria (NL, OM). The PRISMA-P, 2015 statement will be used to guide reporting of the review findings. PRISMA flow will be used to indicate the outcome of article search and selection for inclusion in the review [7]. Strengthening Reporting of Genetic Association Studies (STREGA) guidelines [8] will be used in developing data extraction form as this is a systematic review of molecular outcome(s) (prevalence of *Pfcrt* K76T *and Pfmdr* N86Y). Any disagreement between the two reviewers will be resolved by discussion and any further disagreement referred to a tie-breaker.

### Data abstraction

Three independent reviewers (BB, NL, and OM) will abstract data using a pre-set abstraction form. Data abstraction tool will be developed in Excel spread sheet 2007 (Additional file 3). The tool will be piloted on five (05) articles and adjusted accordingly based on findings of the pilot and the expected review outcomes. Kappa agreement between the reviewers will be calculated and any disagreement resolved by discussion. Any further disagreement will be referred to the tie-breaker. The authors of the reviewed articles will be contacted in case there is need for clarification and or more information not indicated in the article.

### Data items

In this review, data will be sought on the following: source of parasite DNA used, *P*. *falciparum* (*population*), use of chloroquine in malaria treatment (*exposure*), occurrence of mutations PfcrtK76T and Pfmdr1N86Y after change in malaria treatment policy (*outcome*), design of studies used to establish occurrence of chloroquine resistance alleles (*study design*), source of funding for the studies, year when the primary study was done (*time*). There is no pre-planned data assumptions and simplifications.

## Outcomes and prioritization

### Primary review outcome

Prevalence of *Pfcrt* K76T and *Pfmdr1* N86Y alleles among *Plasmodium falciparum* parasites in countries affected by malaria since change in treatment policy from chloroquine to artemisinin agents.

### Secondary outcomes

Duration of time since policy change from chloroquine to artemisinin agents, medicines used in malaria treatment, and method used in the identification of mutation. Prevalence of chloroquine use in malaria treatment after malaria treatment policy change, malaria transmission intensity (nature of malaria transmission), and prevalence of genotypic chloroquine resistance (*Pfcrt K76T* and *Pfmdr1* N86Y) among *P*. *falciparum* parasites the year when malaria treatment policy was changed to artemisinin agents, the medicines used in malaria treatment since change in treatment policy, year when malaria treatment was changed from chloroquine to artemisinin-based combination therapy (ACTs).

With the emerging threat of artemisinin resistance, approaches to identify potential alternative treatment for malaria is urgently needed. Various studies have report existence of reversal to chloroquine resistance in *Plasmodium falciparum* parasites. However, the extent of this reversal across malaria-affected countries in not known. This makes it challenging to evaluate the potential re-emerging role of chloroquine in malaria treatment. Therefore, establishing the main outcome of this review is critical in informing a discussion on the potential re-introduction of chloroquine in malaria treatment. This provides hope especially with the current emergence of artemisinin resistance.

#### Data management

Abstracted data will be kept in Excel spread sheet 2007. Articles for the review will be kept Endnote file by the lead reviewer (MO).

#### Data analysis

Data will be quantitatively synthesized only if there is no high heterogeneity in the main review outcome across included articles. The level of heterogeneity in the articles will be established using *I*^2^ statistic. The *I*^2^ statistic will be used to indicate percentage (%) heterogeneity that can be attributed to between-study variance. Interpretation: *I*^2^ = 25% (small heterogeneity), *I*^2^ = 50% (moderate heterogeneity), *I*^2^ = 75% (large heterogeneity). Narrative assessment of the data will be done. Pooling of data will be done using measures of central tendency (means) and proportions. Strengthening Reporting of Genetic Association studies (STREGA) guideline [8] will be used to develop data extraction form that will be used in extracting data from the included articles. Random effects model will be used. The analysis will be done in STATA 13.0 software. Sub-group analysis will be done (countries/regions, duration of use of artemisinin agents).

#### Publication bias assessment

Publication bias is the influence of research findings on the probability of a study being published. This will be assessed by plotting funnel plots and using the symmetry of the plots to detect likelihood of publication bias among the articles included in the review. The articles will be adjusted for publication bias using trim and fill method [9]. Briefly, the weighted mean effect size for the full data set will be calculated; the number of studies in the outlying part of the plot that is asymmetrical with respect to the mean will be calculated; these studies will then be temporally removed (“trimmed”) and the value of the mean effect re-estimated; the procedure will be repeated for the new mean, after the estimate of the number of studies that need to be trimmed reaches an asymptotic value; the “missing” studies are added as the mirror-image counterparts of the trimmed studies; and the new mean effect size and confidence interval are then re-calculated.

## Discussion

*Plasmodium* parasites have developed resistance to nearly all antimalarial agents that have been introduced in treatment so far. Chloroquine, a previous highly efficacious and affordable antimalarial agent, suffered widespread loss of efficacy in most malaria-endemic countries globally [10]. The high resistance to chloroquine and its subsequent withdrawal from use in the 1990s across most malaria-endemic countries was responsible for more than doubling of malaria-associated mortality especially in Sub-Saharan Africa [11]. Chloroquine was replaced by artemisinin agents; however, unlike chloroquine, these agents are expensive with a high pill burden, a potential barrier to malaria treatment [10]. This in addition to the recent detection of artemisinin resistance in Southeast Asia further supports the need for continued search for an alternative to artemisinin agents in malaria treatment [3, 12]. However, currently, there is limited investment on research leading to discovery and development of new antimalarial agents globally.

*Plasmodium falciparum* parasite which is responsible for majority of malaria cases has recently been reported to have regained susceptibility to chloroquine [13]. This provides hope for malaria treatment and elimination efforts in malaria-affected countries. Although some studies have reported existence of reversal in chloroquine resistance, the extent of this reversal is not known across malaria-endemic countries. It is thus difficult to predict the potential re-emerging role of chloroquine in malaria treatment and eradication efforts. The current review seeks to establish the extent of re-emergence of chloroquine susceptibility among *Plasmodium falciparum* parasites across malaria-endemic countries. This will help policy makers in establishing the potential for re-introduction of chloroquine in malaria treatment across malaria-endemic countries.

However, the potential re-introduction of chloroquine in malaria treatment will require more than knowledge of the extent of reversal in chloroquine resistance. This is especially due to potential re-emergence of resistance among *Plasmodium* parasites upon re-introduction of chloroquine in malaria treatment. In addition, currently, there is no comprehensive information on chloroquine resistance among *Plasmodium falciparum* parasites in malaria-affected countries and is what the current review is seeking to address.

## Additional files

Additional file 1: PRISMA-P 2015 Checklist. (DOC 105 kb)

Additional file 2: PUBMED search. (DOC 24 kb)

Additional file 3: Re-emergence of Chloroquine sensitivity review Data abstraction form. (XLS 27 kb)

## Abbreviations

PRISMA: Preferred Reporting Items for Systematic reviews and Meta-Analysis
PROSPERO: International Prospective Register of Systematic Reviews
STREGA: Strengthening Reporting of Genetic Association Studies
