# Supplementary material for: Prevalence of chloroquine resistance alleles among Plasmodium falciparum parasites in countries affected by malaria disease since change of treatment policy: a systematic review protocol
Source: Syst Rev. 2018 Jul 27;7:108. doi: 10.1186/s13643-018-0780-z (PMC6064057; doi:10.1186/s13643-018-0780-z)
Supplement: Supplementary file 1 — PRISMA-P 2015 Checklist. (DOC 105 kb) [file 13643_2018_780_MOESM1_ESM.doc]

**PRISMA-P 2015 Checklist**

# ****This checklist has been adapted for use with systematic review protocol submissions to BioMed Central journals from Table 3 in Moher D et al**:** Preferred reporting items for systematic review and meta-analysis protocols (PRISMA-P) 2015 statement. *Systematic Reviews* **2015 4:1**

# An Editorial from the Editors-in-Chief of *Systematic Reviews* details why this checklist was adapted - **Moher D, Stewart L & Shekelle P:** Implementing PRISMA-P: recommendations for prospective authors. *Systematic Reviews* 2016 **5**:15

| **Section/topic** | **#** | **Checklist item** | **Information reported** | | **Line number(s)** |
| --- | --- | --- | --- | --- | --- |
|  |  |  | **Yes** | **No** |  |
| **ADMINISTRATIVE INFORMATION** | | | | | |
| **Title** | | | | | |
| Identification | 1a | Identify the report as a protocol of a systematic review | / **Yes** |  | 02, 43, 94, 204, 207, 218 & 219 |
| Update | 1b | If the protocol is for an update of a previous systematic review, identify as such |  | / **No** | Not applicable |
| **Registration** | 2 | If registered, provide the name of the registry (e.g., PROSPERO) and registration number in the Abstract | / **Yes** |  | 46, 47, 98, 99 & 100 |
| **Authors** | | | | | |
| Contact | 3a | Provide name, institutional affiliation, and e-mail address of all protocol authors; provide physical mailing address of corresponding author | / **Yes** |  | 4 –to- 24 |
| Contributions | 3b | Describe contributions of protocol authors and identify the guarantor of the review | / **Yes** |  | 257 - 259 |
| **Amendments** | 4 | If the protocol represents an amendment of a previously completed or published protocol, identify as such and list changes; otherwise, state plan for documenting important protocol amendments |  | / **No** | Not applicable |
| **Support** | | | | | |
| Sources | 5a | Indicate sources of financial or other support for the review | / Yes |  | 249 - 255 |
| Sponsor | 5b | Provide name for the review funder and/or sponsor | / Yes |  | 249 - 255 |
| Role of sponsor/funder | 5c | Describe roles of funder(s), sponsor(s), and/or institution(s), if any, in developing the protocol | / Yes |  | 254, 255 |
| **INTRODUCTION** | | | | | |
| **Rationale** | 6 | Describe the rationale for the review in the context of what is already known | / Yes |  | 75 – 82 & 177 - 184 |
| **Objectives** | 7 | Provide an explicit statement of the question(s) the review will address with reference to participants, interventions, comparators, and outcomes (PICO) | / Yes |  | 87 -89 |
| **METHODS** | | | | | |
| **Eligibility criteria** | 8 | Specify the study characteristics (e.g., PICO, study design, setting, time frame) and report characteristics (e.g., years considered, language, publication status) to be used as criteria for eligibility for the review | / Yes |  | 103-120 |
| **Information sources** | 9 | Describe all intended information sources (e.g., electronic databases, contact with study authors, trial registers, or other grey literature sources) with planned dates of coverage | / Yes |  | 122 - 128 |
| **Search strategy** | 10 | Present draft of search strategy to be used for at least one electronic database, including planned limits, such that it could be repeated | / Yes |  | 130 – 135 |
| ***STUDY RECORDS*** | | | | | |
| Data management | 11a | Describe the mechanism(s) that will be used to manage records and data throughout the review | / Yes |  | 186 - 187 |
| Selection process | 11b | State the process that will be used for selecting studies (e.g., two independent reviewers) through each phase of the review (i.e., screening, eligibility, and inclusion in meta-analysis) | /  Yes |  | 140 – 143  145 – 151 |
| Data collection process | 11c | Describe planned method of extracting data from reports (e.g., piloting forms, done independently, in duplicate), any processes for obtaining and confirming data from investigators | / Yes |  | 145 – 151 |
| **Data items** | 12 | List and define all variables for which data will be sought (e.g., PICO items, funding sources), any pre-planned data assumptions and simplifications | / Yes |  | 153 – 158 |
| **Outcomes and prioritization** | 13 | List and define all outcomes for which data will be sought, including prioritization of main and additional outcomes, with rationale | / Yes |  | 162 – 165, 167 – 175, 177 - 184 |
| **Risk of bias in individual studies** | 14 | Describe anticipated methods for assessing risk of bias of individual studies, including whether this will be done at the outcome or study level, or both; state how this information will be used in data synthesis | / Yes |  | 200 - 204 |
| ***DATA*** | | | | | |
| **Synthesis** | 15a | Describe criteria under which study data will be quantitatively synthesized | / Yes |  | 1891-190 |
|  | 15b | If data are appropriate for quantitative synthesis, describe planned summary measures, methods of handling data, and methods of combining data from studies, including any planned exploration of consistency (e.g., *I* ^2^, Kendall’s tau) | / Yes |  | 191 – 198  203 - 204 |
|  | 15c | Describe any proposed additional analyses (e.g., sensitivity or subgroup analyses, meta-regression) | / Yes |  | 197 - 198 |
|  | 15d | If quantitative synthesis is not appropriate, describe the type of summary planned | / Yes |  | 194 |
| **Meta-bias(es)** | 16 | Specify any planned assessment of meta-bias(es) (e.g., publication bias across studies, selective reporting within studies) | / Yes |  | 200 - 204 |
