# Supplementary material for: Prevalence of chloroquine resistance alleles among Plasmodium falciparum parasites in countries affected by malaria disease since change of treatment policy: a systematic review protocol
Source: Syst Rev. 2018 Jul 27;7:108. doi: 10.1186/s13643-018-0780-z (PMC6064057; doi:10.1186/s13643-018-0780-z)
Supplement: Supplementary file 2 — PUBMED search. (DOC 24 kb) [file 13643_2018_780_MOESM2_ESM.doc]

**PUBMED search**

((((((Chloroquine OR 4-aminoquinoline OR Amodiaquine OR Piperazine))) AND ((“Plasmodium falciparum” OR Parasite OR Parasites OR “Malaria parasites” “Malaria parasite” OR Malaria OR Fever OR Plasmodium))) AND ((Prevalence OR Occurrence OR Proportion OR Spread OR Frequency OR Extent))) AND ((Resistance OR “Resistance alleles” OR “Resistance mutations” OR Polymorphisms OR “Resistance reversal” OR “Pfcrt K76T” OR “Pfmdr1 N86Y” OR “Resistance reversal” OR Mutations OR “Parasite sensitivity” OR Sensitivity OR Susceptibility OR “Parasite susceptibility”))) AND ((Afghanistan OR Algeria OR Angola OR Bangladesh OR Belize OR Benin OR Bhutan OR “Bolivia (Plurinational State of Bolivia)” OR Botswana OR Brazil OR “Burkina Faso” OR Burundi OR Cambodia OR Cameroon OR “Cape Verde” OR “Central African Republic” OR Chad OR China OR Colombia OR Comoros OR Congo OR “Côte d'Ivoire” OR “Ivory Coast” OR “Democratic People's Republic of Korea” OR “Democratic Republic of the Congo” OR Djibouti OR “Dominican Republic” OR Ecuador OR “El Salvador” OR “Equatorial Guinea” OR Eritrea OR Ethiopia OR “French Guiana” OR Guyana OR Gabon OR Gambia OR Ghana OR Guatemala OR Guinea OR “Guinea-Bissau” OR Guyana OR Haiti OR Honduras OR India OR Indonesia OR “Iran (Islamic Republic of Iran)” OR Kenya OR “Lao People's Democratic Republic” OR Liberia OR Madagascar OR Malawi OR Malaysia OR Mali OR Mauritania OR Mexico OR Mozambique OR Myanmar OR Namibia OR Nepal OR Nicaragua OR Niger OR Nigeria OR Pakistan OR Panama OR “Papua New Guinea” OR “Peru” OR Philippines OR “Republic of Korea” OR Rwanda OR “Sao Tome and Principe” OR “Saudi Arabia” OR Senegal OR “Sierra Leone” OR “Solomon Islands” OR “Somalia” OR “South Africa” OR “South Sudan” OR Sudan OR Suriname OR Swaziland OR Thailand OR “Timor-Leste” OR Togo OR Uganda OR “United Republic of Tanzania (Mainland)” OR “United Republic of Tanzania (Zanzibar)” OR “Vanuatu” OR “Venezuela (Bolivarian Republic of Venezuela)” OR “Viet Nam” OR Yemen OR Zambia OR Zimbabwe OR Peru OR Yemen OR Paraguay OR “Costa Rica” OR Belize OR “Tropical countries” OR Tropics)) Filters: Publication date from 1990/01/01 to Current; Humans
